# Model-based analysis on social acceptability and feasibility of a focused protection strategy against the COVID-19 pandemic

**DOI:** 10.1038/s41598-021-81630-9

**Published:** 2021-01-21

**Authors:** Takashi Akamatsu, Takeshi Nagae, Minoru Osawa, Koki Satsukawa, Takara Sakai, Daijiro Mizutani

**Affiliations:** 1 Graduate School of Information Sciences, Tohoku University, Sendai, Miyagi 980-8579 Japan; 2 Graduate School of Engineering, Tohoku University, Sendai, Miyagi 980-8579 Japan; 3 Institute of Economic Research, Kyoto University, Kyoto, 606-8501 Japan; 4 New Industry Creation Hatchery Center, Tohoku University, Sendai, Miyagi 980-8579 Japan; 5 International Research Institute of Disaster Science, Tohoku University, Sendai, Miyagi 980-8572 Japan

**Keywords:** Differential equations, Dynamical systems, Population dynamics, Systems analysis

## Abstract

This paper studies the social acceptability and feasibility of a focused protection strategy against coronavirus disease 2019 (COVID-19). We propose a control scheme to develop herd immunity while satisfying the following two basic requirements for a viable policy option. The first requirement is social acceptability: the overall deaths should be minimized for social acceptance. The second is feasibility: the healthcare system should not be overwhelmed to avoid various adverse effects. To exploit the fact that the disease severity increases considerably with age and comorbidities, we assume that some focused protection measures for those high-risk individuals are implemented and the disease does not spread within the high-risk population. Because the protected population has higher severity ratios than the unprotected population by definition, the protective measure can substantially reduce mortality in the whole population and also avoid the collapse of the healthcare system. Based on a simple susceptible-infected-recovered model, social acceptability and feasibility of the proposed strategy are summarized into two easily computable conditions. The proposed framework can be applied to various populations for studying the viability of herd immunity strategies against COVID-19. For Japan, herd immunity may be developed by the proposed scheme if ${\mathcal {R}}_0 \le 2.0$ and the severity rates of the disease are 1/10 times smaller than the previously reported value, although as high mortality as seasonal influenza is expected.

## Introduction

The coronavirus disease 2019 (COVID-19) pandemic has been a huge global health threat, with over 10 million cases and 500,000 deaths confirmed worldwide as of June 30, 2020^1^. From the experiences of China, Italy, and the United States, it has been observed that COVID-19 can overwhelm healthcare capacities. Because of the absence of an effective antiviral drug or vaccine, many countries have adopted non-pharmaceutical interventions (NPIs), such as closing schools and workplaces and imposing rigorous social distancing measures, to reduce transmission of the virus. Initial efforts by governments worldwide have been concentrated on the short-run *suppression* of the first wave of the pandemic to avoid the collapse of the healthcare system. Suppression of an outbreak can, however, leave a large portion of the population uninfected and susceptible; therefore, resurgence of the outbreaks as severe as the initial one becomes a possibility^2–4^. Moreover, radical NPIs aimed at suppression are not sustainable, as they have already had devastating impacts on the global economy and personal lives of many. Long-run strategies that go beyond simple relaxation of the initial suppression-oriented responses are crucial, as we probably are at least one or two years away from substantial supply of a vaccine. Continued circulation of the virus in the global population for a prolonged period seems to be inevitable. A possible option might be the development of *herd immunity* by ensuring the presence of a sufficiently high proportion of immune individuals in the population; it may be achieved through a carefully managed, slow spread of infection, which could prevent the collapse of the healthcare system^5^.

Social acceptance is a significant issue for all types of herd immunity policies because it is associated with a considerably large number of infections and hence high mortality. For an infectious disease with a basic reproduction number ${\mathcal {R}}_0 > 1$, herd immunity requires that at least a proportion $1 - {\mathcal {R}}_0^{-1}$ of the entire population be infected. For COVID-19, the basic reproduction number is reported to be around 2.5^6–8^, whereas the infection fatality ratio (IFR) is reported to be around 0.6 to 0.7%^9–11^. A crude estimate on the basis of these numbers is that the resulting mortality during the progress toward herd immunity can reach 400 per 100,000 population, that is, about 500,000 deaths in Japan and 1.3 million deaths in the United States. These figures would not be socially acceptable, given that the estimated deaths related to seasonal influenza in Japan and the United States were around 10,000 and 34,000 in the 2018–2019 season, respectively^12^. The basic premise of our research is that the “true” IFR for COVID-19 is lower than the reported value, as no herd immunity approaches can obtain social acceptance otherwise.

We propose a long-run control strategy that aims at developing herd immunity and study its social *acceptability* (in terms of the resulting mortality) and *feasibility* (in terms of healthcare capacity). The proposed control strategy includes two components. The first component is a focused protection measure for high-risk individuals, e.g., the elderly and those with underlying health conditions), who are likely to die if they contract COVID-19. The second component is a dynamic control measure (time-dependent reductions in social activities) among the unprotected to keep the daily number of infected individuals below the healthcare capacity, thereby avoiding the collapse of the healthcare system. Under the focused protection of high-risk individuals, the average disease severity ratios in the unprotected population becomes much smaller than those of the whole population including the vulnerable. Thus, the focused protection has two-fold implications. First, it contributes to social acceptance because it can substantially reduce mortality. Second, it contributes to feasibility because the society can accept a larger number of infections than it can without focused protection, as the proportion of severe cases goes down under the protective measure. With these effects and the imposed dynamic control measure, the number of infected individuals can increase without causing a collapse of the healthcare system. For COVID-19, age is a key factor associated with disease severity, with fatality increasing disproportionately with age. For instance, those aged $\ge 80$ years are 1000 to 10,000 times more likely to die from infection than are those aged $< 20$ years, according to previous estimates^9,11^. On the basis of this fact, our numerical examples consider an age-based definition of the protected group. Our analyses reveal that if several conditions are met with the basic reproduction number of the epidemic, ${\mathcal {R}}_0$, and several other characteristic parameters of the system, the whole population can acquire herd immunity without the collapse of the healthcare system, with a substantial reduction in mortality.

Rather than simulating highly structured models, we build on the basic susceptible-infected-recovered (SIR) model^13^. Further, as an idealization, our mathematical model assumes that the disease does not spread among the protected group at all, i.e., the group is isolated from the SIR epidemic dynamics during the outbreak. At the cost of the simplification, we can study the feasibility and social acceptability of herd immunity strategies semi-analytically with a limited number of input variables. The only required inputs are the age composition of the population, the healthcare system’s service capacity, and the age-specific severity rates for COVID-19; we acknowledge that uncertainties remain for the last parameter. For its simplicity, the proposed framework for investigating social acceptability and feasibility of focused protection policies is universally applicable to different populations without resorting to data-intensive and model-specific structural simulations and calibrations. For Japan, as an example, herd immunity can be developed if ${\mathcal {R}}_0 \le 2.0$ and the severity rates of the disease (including the IFR) is 1/10 times lower than the previously reported value^9^. Numerical results obtained by the proposed framework can be easily updated by incorporating the latest estimates for the input parameters.

## Method

### Control scheme

Consider a population that faces a novel infectious disease with a basic reproduction number ${\mathcal {R}}_0 > 1$. We propose a control scheme that consists of the two components discussed below. See Supplementary Information for the mathematical details.

**Figure 1:**
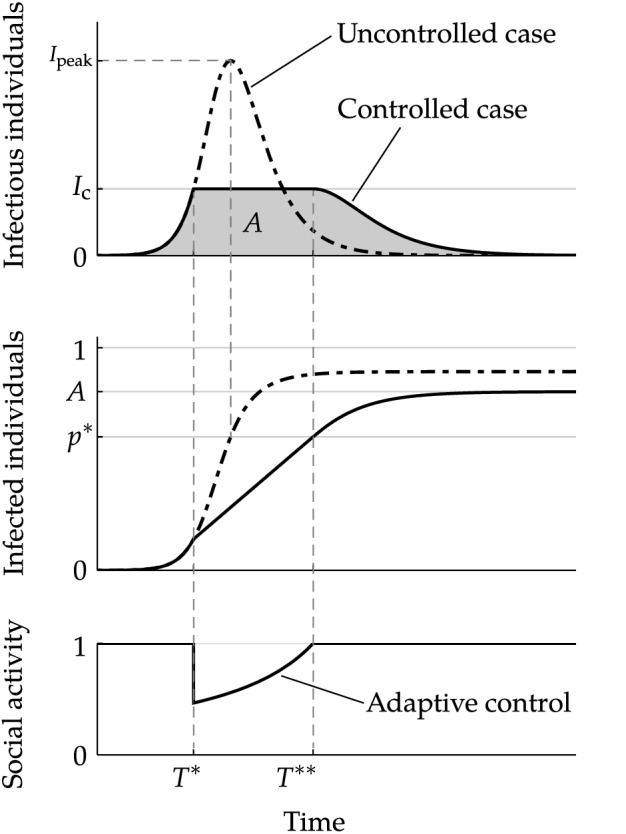
Proposed adaptive control measure. The top and middle shows the number of infectious individuals *I*(*t*) and infected (infectious or recovered) individuals, respectively, $$1 - S(t)$$ at time *t*. An uncontrolled case is indicated using the dot-dashed curve, whereas a controlled case is indicated using the black solid curve. An adaptive control that reduces social activity as in the bottom panel is imposed at $$T^*$$ and lifted when herd immunity in this group is established at $$T^{**}$$. The area of the gray region below the controlled curve in the top panel presents the final number of infected individuals in the active group, *A*. As the number of infectious individuals start to decrease at $$T^{**}$$, *A* will exceed $$p^* = 1 - {\mathcal {R}}_0^{-1}$$.

#### Protection measure for high-risk individuals

To minimize the fatality from an outbreak, some protective measures for high-risk individuals must be implemented^14^. Existing evidence consistently suggests that age is a key factor that affects the severity of COVID-19, with the fatality ratio substantially increasing among elderly individuals^9,11^. We assume that the entire population is partitioned into two groups: the *protected* and the *active* groups. The former group consists of high-risk individuals, such as elderly individuals and those with pre-existing medical conditions, who are likely to die if they contract COVID-19. The latter consists of low-risk individuals who have a high probability of recovering from the infection. The former group is safely isolated from the active group and protected from the disease during the outbreak. Individuals in the latter group can interact with others in the same group and thus are susceptible to infection. The size of the active group is normalized to unity. The size of the entire population, including both the protected and the active groups, is denoted by $N \ge 1$. The relative size of the active group in the entire population, $n\equiv 1/N \in (0,1]$, is chosen prior to the dynamic control measure described below.

#### Adaptive control measure for low-risk individuals

As the disease can spread within the active group, some dynamic control measures should be implemented in this group to avoid the collapse of the healthcare system. Figure 1 illustrates the adaptive control scheme. Let *I*(*t*) be the number of infectious individuals in the active group at time *t*. To keep *I*(*t*) below the maximum level that the healthcare system can handle, the control scheme has a predetermined threshold $I_{\rm c}$ for *I*(*t*) and aims to maintain

1 $$\begin{aligned} I(t) \le I_{\rm c}. \end{aligned}$$

To achieve this, the social activity level at *t* is set to

2 $$\begin{aligned} \alpha (t) = \frac{1}{{\mathcal {R}}_0 S(t)} \end{aligned}$$

once *I*(*t*) reaches $$I_{\rm c}$$ at some time $$T^*$$, as shown in the bottom panel of Fig. 1. By this adaptive control, the effective reproduction number $$\alpha (t) {\mathcal {R}}_0 S(t)$$ is kept at 1, so that *I*(*t*) stays at the threshold level $$I_{\rm c}$$ during the peak period (the top panel of Fig. 1). At some time $$T^{**} > T^*$$, the remaining number of susceptible individuals equals $${\mathcal {R}}_0^{-1}$$, or the cumulative number of infected individuals equals

3 $$\begin{aligned} p^*\equiv 1 - {\mathcal {R}}_0^{-1}. \end{aligned}$$

The proportion $$p^*\in (0,1)$$ is the well-known *herd immunity threshold*, or the threshold share of infected individuals for a population to develop herd immunity^15^. Thus, herd immunity *for the active group* (not necessarily for the whole population, including the protected group) is developed at $$T^{**}$$. Without any control, the number of infectious individuals in the active group declines after $$T^{**}$$ because of herd immunity. When the outbreak within the active group ends, the protective measures among high-risk individuals can be lifted. The area below the epidemic curve in the top panel of Fig. 1 corresponds to the final number of recovered individuals, which we denote as *A*.

### Social acceptability and feasibility

There are three conditions under which the proposed control policy can be a viable policy option against a novel infectious disease such as COVID-19. First, for social acceptance, the overall mortality should not become too large. Second, the healthcare system should not be overwhelmed. Third, the final number of infected individuals, *A*, should be sufficiently high to acquire herd immunity. The last two conditions may be called feasibility conditions. If the proposed strategy is both socially acceptable and feasible, herd immunity approaches of the proposed type would be viable policy options.

#### Social acceptability: upper bound for the overall mortality

The first component (a) of the control scheme aims at reducing mortality. Suppose that the society can accept only $\overline{N} < N$ lost lives owing to the epidemic, then the following condition must be met:

4 $$\begin{aligned} \phi A \le \overline{N}, \end{aligned}$$

where $\phi \in (0,1)$ is the average mortality rate in the active group on infection and *A* is the final cumulative number of infections in the active group. The left-hand side of (4) is the number of lives lost by the end of the outbreak. Thus, the proposed policy is (*socially*) *acceptable* if condition (4) is met.

The condition (4) restricts the size of the active group compared with that of the whole population. The larger the active group, the more high-risk individuals are included in it. Thus, $\phi$ increases with the size of the active group. Evidence suggests that the fatality ratio for the elderly is by far higher than that for the young^9^. The flip side of this is that we can substantially reduce the average mortality rate $\phi$ for the active group by imposing the protection measure (a), thereby relaxing (4) without the difficult compromise of increasing $\overline{N}$ in the first place.

#### Feasibility (1): healthcare system’s capacity

The second component (b) of the proposed control scheme aims at acquiring herd immunity with limited healthcare resources. The first feasibility requirement for this component is that the control threshold $I_{\rm c}$ must be lower than the *effective capacity* of the healthcare system, which we denote by $I_{\max }$, for the instantaneous number of infectious individuals. For example, let $\mu > 0$ denote the per capita beds available for the care of severe cases that require hospitalization, i.e., the *service capacity* of the healthcare system. To avoid various adverse effects, including a surge in mortality, the service capacity must not be exceeded. Suppose that a given proportion, $\theta \in (0,1)$, of infected individuals in the active group needs hospitalization. Then, for the healthcare system to maintain its normal functioning, we must require $\theta I_{\rm c} \le N \mu$, i.e.,

5 $$\begin{aligned} I_{\rm c} \le I_{\max } = \frac{N \mu }{\theta }. \end{aligned}$$

Thus, $I_{\max } = N \mu /\theta$ gives the effective capacity. If $\theta$ is the proportion of infected individuals who need critical care, then the service capacity $\mu$ should be replaced with the per capita intensive care unit (ICU) beds.

The effective capacity $I_{\max }$ is decreasing in $\theta$ and thus is minimized when no protection measure for high-risk individuals is in place. Equivalently, $I_{\max }$ is increasing in *N* because $\theta$ is decreasing in *N*. Furthermore, $I_{\max }$ is increasing in *N* because the per capita service capacity *for the active group* is $N \mu$. Therefore, by protecting the elderly and other high-risk individuals, we can considerably increase $I_{\max }$ without enhancing the baseline service capacity $\mu$.

#### Feasibility (2): acquisition of herd immunity

The last condition requires that the final number of infected individuals in the active group, *A*, must be sufficiently high to acquire herd immunity for the whole population. Specifically, *A* should satisfy the following condition for herd immunity *as the whole population* to be acquired:

6 $$\begin{aligned} A \ge p^* N, \end{aligned}$$

where we recall that $p^* = 1 - {\mathcal {R}}_0^{-1}\in (0,1)$ is the herd immunity threshold. If (6) is met, the protection measure for high-risk individuals can be lifted after a sufficient decrease in the number of infected individuals in the active group without causing a secondary outbreak.***

**Figure 2:**
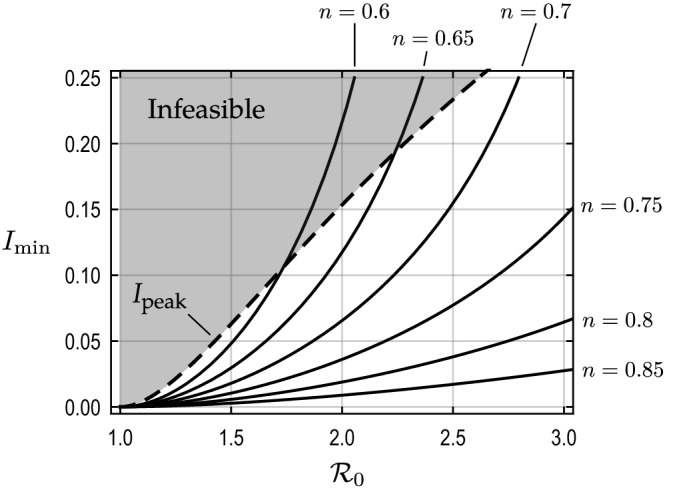
Minimum level of the control threshold to acquire herd immunity with varying shares of the active group in the whole population, $n = 1/N \in (0,1]$. For each *n*, the condition (6) is satisfied if $I_{\rm c} \ge I_{\min }$. The proposed strategy with any control threshold, $I_{\rm c}$, that is above the black solid curve can achieve herd immunity as the whole population. The dashed curve indicates the peak number of infectious individuals $I_{\rm peak}$, which is an increasing function of ${\mathcal {R}}_0$, in the uncontrolled scenario. The assumed control scheme is feasible (in terms of the condition (6) if $)I_{\min } \le I_{\rm peak}$.

From the middle panel of Fig. 1, we note that *A* is an increasing function of $I_{\rm c}$ so long as $I_{\rm c} \le I_{\rm peak}$. Then, because *A* must be sufficiently high, there is a minimum value $I_{\min }$ for $I_{\rm c}$ so that the whole population can acquire herd immunity if $I_{\rm c} \ge I_{\min }$. The threshold, $I_{\min }$, is an increasing function of ${\mathcal {R}}_0$ and *N*, as the right-hand side of (6) is increasing in both ${\mathcal {R}}_0$ and *N*. See Supplementary Information for the concrete formula of $I_{\min }$. Figure 2 shows the minimum level $I_{\min }$ against ${\mathcal {R}}_0$ for different choices of $n = 1/N\in (0,1]$. Because $I_{\rm c}$ cannot exceed the peak level for the uncontrolled scenario, the assumed control scheme is feasible only if $I_{\min } \le I_{\rm peak}$ (see Fig. 1).

Using the two conditions (5) and (6), the proposed control requires $I_{\rm c}$ to satisfy $I_{\min } \le I_{\rm c} \le I_{\max }$ and $I_{\rm c} \le I_{\rm peak}$. Thus, it must be that

7 $$\begin{aligned} I_{\min } \le \min \left\{ I_{\rm peak}, I_{\max }\right\} . \end{aligned}$$

We say the proposed strategy is *feasible* if (7) is met.

#### Summary of the variables

The variables that appear in our framework are as follows. The policy variables that characterize the proposed control scheme are the size of the active group $n = 1/N$ and the control threshold $I_{\rm c}$. The environmental constants related to the disease, which cannot be modified by the policy, are ${\mathcal {R}}_0$, $\theta$, and $\phi$, where the latter two are increasing functions of *n*. The other constants are the per capita healthcare service capacity $\mu$, which can be increased in the long run, and the socially acceptable mortality $\overline{N}$.

## Numerical results

Considering Japan as an example, this section illustrates how we can assess the social acceptability and feasibility of the proposed control scheme. We can define the protected group arbitrarily up to availability of group-specific disease severity data. With age being a key factor for the severity of COVID-19, a pragmatic method of defining the active group would be selecting a threshold age $a^*$, whereby individuals with age $< a^*$ constitute the active group. To obtain $\theta$ and $\phi$ numerically as functions of $a^*$, we simply combine an available estimate for the age-specific infection hospitalization ratio (IHR) and infection fatality ratio (IFR) for COVID-19^9^ and the latest estimate for the population composition of the country^16^.

Table 1 summarizes the basic parameters *n*, $\theta$, and $\phi$ for the active group with different choices of $a^*$. Case 0 is the uncontrolled scenario without any protection or dynamic control measures. Cases 1, 2, 3, and 4 correspond to the proposed control schemes with $a^*=50$, 55, 60, and 65. As observed, both the IHR $\theta$ and IFR $\phi$ increases with $a^*$. Both IHR and IFR are substantially lower for Cases 1 to 4 than for Case 0 (no protection). Below, we examine the social acceptability and feasibility conditions using the baseline parameters shown in the table.

**Table 1:** Infection hospitalization ratio and infection fatality ratio for the active group with different $a^*$ under the Japanese population composition. The age-specific severity ratios are adopted from^9^, whereas the Japanese population data from^16^. Case 0 (no protection measure) is shown for reference.

	Case 0	Case 1	Case 2	Case 3	Case 4
Age threshold $a^*$	$\infty$	50	55	60	65
The active group	All individuals	0–49	0–54	0–59	0–64
$n = 1/N$	1	0.52	0.59	0.65	0.71
Infection hospitalization ratio $\theta$ (%)	7.5	2.1	2.8	3.3	4.0
Infection fatality ratio $\phi$ (%)	1.9	0.066	0.13	0.17	0.34

### Social acceptability

We observe that the proposed approach, or herd immunity strategies in general, would not be socially acceptable for Japan if the reported estimate of IFR for COVID-19 is not an overestimation. Table 2 shows the minimum mortality that results owing to the proposed herd immunity strategy in Cases 1 to 4 under different values of ${\mathcal {R}}_0$. The minimum mortality is obtained simply by multiplying the Japanese population by $\phi p^*$, where $p^* = 1 - {\mathcal {R}}_0^{-1}$ is the herd immunity threshold. The condition (4) requires that the resulting mortality should not be too high. Table 2 clearly indicates that herd immunity approaches are not socially acceptable if the reported IFR is close to the “true” value. For example, ${\mathcal {R}}_0 = 2.5$ must result in at least 130,000 deaths in Case 3. Although it is approximately 1/10 times smaller than the uncontrolled scenario with 1.4 million deaths (Case 0), such a number would not be socially acceptable for a country with less than 1000 confirmed COVID-19 deaths (as of 15 May 2020). In particular, Case 4 would not become socially acceptable for any ${\mathcal {R}}_0$ shown in the table.

That said, if the reported IFR is true, *any form of* herd immunity strategies would not be socially acceptable for the country. However, the adopted baseline estimate of the IFR is a pessimistic bound. For instance, various reports suggest that there is a large number of asymptomatic infections^17–19^, which can imply $\theta$ and $\phi$ are smaller than the baseline estimates. For Japan, a recent study based on serological testing argued that there might have been approximately 400- to 850-fold infections more than the confirmed cases with PCR testing in Kobe City, Japan^20^. This result could be an overestimation, with all its limitations, including the specificity of the employed test kit and selection bias. However, these studies consistently suggest that true IFR can be much smaller than the previous estimates owing to asymptomatic or mild infections.

Below, as a thought experiment, we assume that the IFR $\phi$ (and also the IHR $\theta$ for consistency) is 1/10 times smaller than the reported estimate by Verity et al.^9^. Based on the assumption that the true IFR of COVID-19 is at least 1/10 times smaller than the reported value, the resulting mortality owing to the proposed strategy becomes the order of 10,000 for Japan (Case 3), which is akin to the average annual influenza-related mortality in the country.

**Table 2:** The minimum mortality to acquire herd immunity (thousand deaths). A blank cell indicates that the share of the active group *n* for that settings is too small to develop herd immunity for the whole population (i.e., $n < p^*$).

${\mathcal {R}}_0$	$p^*$	Case 0	Case 1	Case 2	Case 3	Case 4
1.5	0.41	790	27	53	72	140
2.0	0.50	1200	41	80	110	210
2.5	0.60	1400			130	260

### Feasibility

Next, we evaluated feasibility by considering Case 3 ($a^* = 60$) as an example. Based on a report from the Ministry of Health, Labor and Welfare, we assume that the hospitals’ bed count available for COVID-19 patients in Japan is $3.1 \times 10^4$, or $\mu = 2.5 \times 10^{-4}$ per capita^21^. The effective capacity is then given by

8 $$\begin{aligned} I_{\max } = \frac{N \mu }{\theta } = \frac{\mu }{n\theta } = \frac{2.5 \times 10^{-4}}{n \theta }. \end{aligned}$$

The effective capacity becomes a similar value even if we use the proportion of infections that require critical care for $\theta$ and replace $\mu$ with the per capita-free ICU beds (see Supplementary Information). Table 3 shows the values of the effective capacity for Cases 0 to 4, where $\theta$ is assumed to be 1/10 times the adopted IHR^9^ to ensure consistency with the corresponding assumption on $\phi$. We observe that the protection measure substantially increases $I_{\max }$ in all Cases 1 to 4 compared with Case 0. For example, in Case 3, $I_{\max }$ is approximately 3.4 times greater in Case 3 than in Case 0.

**Table 3:** Effective healthcare capacity $I_{\max }.$

	Case 0	Case 1	Case 2	Case 3	Case 4
$I_{\max }$	0.0330	0.221	0.147	0.113	0.0857

**Figure 3:**
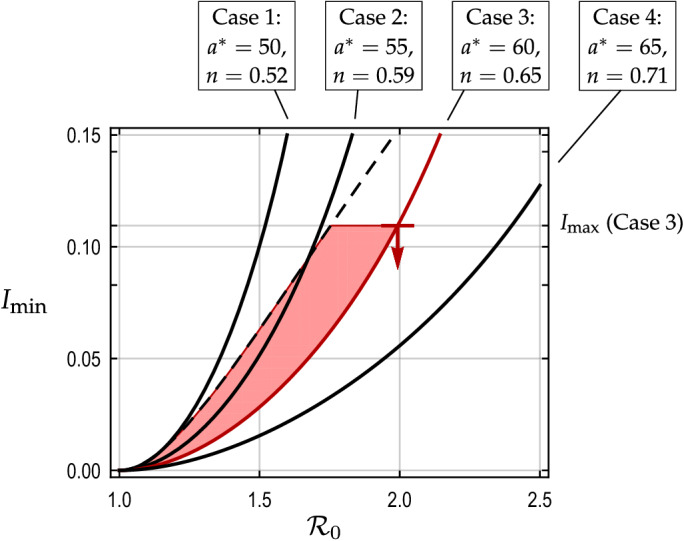
solid black curves show $I_{\min }$ for Cases 1 to 4. The dashed black curve shows $I_{\rm peak}$. The marker indicates $I_{\max }$ for Case 3, below which the proposed control scheme is feasible in terms of the healthcare system capacity. In Case 3, the proposed control scheme is feasible when ${\mathcal {R}}_0$ and $I_{\rm c}$ lie in the shaded region. The feasibility condition (7) is thus satisfied for ${\mathcal {R}}_0 \le 2.0$ in Case 3.

Figure 3 examines the feasibility of the proposed control scheme for Case 3. The solid black lines indicate $I_{\min }$ for different $a^*$, which are obtained using the corresponding shares *n* of the active group (Table 1). Cases 1, 2, and 4 are also shown for comparison. The dashed curve indicates $I_{\rm peak}$ as in Fig. 2, while the marker on $I_{\min }$ in Case 3 shows $I_{\max }$ for that case. The condition (7) means that the proposed control scheme is feasible if $I_{\min }$ stays below both $I_{\rm peak}$ and $I_{\max }$. For Case 3, $I_{\max } = 0.113$ and feasibility in terms of the healthcare capacity is satisfied for ${\mathcal {R}}_0 \le 2.0$. As we see $I_{\max } \le I_{\rm peak}$ when ${\mathcal {R}}_0 \le 2.0$, Case 3 satisfies the condition (7) and hence is feasible if ${\mathcal {R}}_0 \le 2.0$. If ${\mathcal {R}}_0 > 2.0$ is the case, a substantial increase in the hospital beds would in order unless $\theta$ is smaller than our assumption.

## Discussion

This study provides a simple framework for assessing the viability of herd immunity strategies as policy options against COVID-19. We propose a control strategy with a protection measure for the elderly and other high-risk individuals. Using the protection measure, the proposed control scheme aims to (i) minimize the resulting mortality and (ii) prevent the collapse of the healthcare system by reducing the proportion of severe cases during the outbreak. The thought experiment with Japanese parameters suggests that the proposed strategy can be an acceptable and feasible option if the “true” severity of the disease (i.e., the IHR $\theta$ and IFR $\phi$) is lower than the value reported by Verity et al.^9^. In concrete terms, if the severity of COVID-19 is 1/10 times lower than the reported estimate, the proposed control scheme can become a viable policy option when ${\mathcal {R}}_0 < 2.0$.

Besides our numerical examples, which can easily be updated and corrected using the latest data, an important qualitative observation is that protection measures for high-risk individuals are the key to the social acceptability and feasibility of herd immunity approaches. For example, even if the final number of infected individuals is the same, the resulting mortality is considerably reduced by imposing a protection measure (Table 2). Likewise, without increasing the baseline service capacity (hospital beds), the effective capacity of the healthcare system $I_{\max }$ significantly increases through protection measures (Table 3). These results are based on the fact that the severity of COVID-19 increases disproportionately with age. Therefore, it is expected that relatively young populations may be able to acquire herd immunity without causing too many deaths per capita^22^. Furthermore, whether we aim at herd immunity or not, some focused protection measures for high-risk individuals can substantially relax healthcare capacity constraints. Although our numerical examples focus on age as the criteria to define the active and protected groups, our approach can be adjusted to meet the specific situation of the population of interest. For example, the malnourished segment of a developing society may be considered a low immunity group even when the segment is relatively young, and thus should be included in the protected group regardless of age. Identifying appropriate partitions of the population on the basis of local conditions is important to help countries to design effective targeted interventions to protect vulnerable individuals and reduce the burden on healthcare systems^23^.

The proposed control scheme reduces the resulting mortality by minimizing the “overshoot” from the herd immunity threshold for the whole population (we aim at $A = p^* N$). The dynamic control measure considered in this study, which primarily aims to develop herd immunity, is conceptually different from that in previous studies that focused on suppression in the short run. For example, some studies consider intermittent lock-down strategies that aim to keep the number of critically ill patients below the ICU service capacity^3,24^. Because simple suppression policies do not aim at focused protection of high-risk individuals, they face a considerably smaller effective capacity ($I_{\max } = 0.0330$ for Case 0 while $I_{\max } = 0.113$ for Case 3 in Table 3). Therefore, even if herd immunity possibly develops under intermittent suppression without a protection measure for high-risk individuals^24^, the duration of such a control strategy becomes by far longer than that with protection, thereby placing a substantial burden on the economy and also resulting in high mortality. Using more complex age-structured epidemic models, several previous researches explore effectiveness of age-targeted measures in reducing adverse effects in the population^14,25,26^; instead, by focusing on a simple model without age structure, our framework can provide estimates that depend on small number of basic parameters. Reliable estimates for the number of infected individuals in the population are crucial for implementing an adaptive control scheme as the proposed one and assessing how close it is to herd immunity. The introduction of systematic tests at scale would be of high priority^5,27,28^. It will also provide better estimates for disease severity.

Our mathematical model is a stylized simplification of any reality, and thus has several apparent limitations, of which we highlight the following three.

First, we assume the basic SIR model with homogeneous agents as the epidemic dynamics, which may overestimate the herd immunity threshold $p^*$. Individual heterogeneity in susceptibility, which must exist in the real world, is known to reduce the herd immunity threshold substantially^29–31^. In this respect, our model considers a worst-case scenario because we would need a smaller number of infections than that assumed in this paper. Reduction of the required number of infections to achieve herd immunity can also contribute to minimizing the possible social burden of prolonged isolation of high-risk individuals. For asking further important questions, e.g., how long does it take for the population to acquire herd immunity, we would need age-structured epidemic dynamics models^14,25,26^ that take into account individual heterogeneity in susceptibility.

Second, the implementation of protective measures for high-risk individuals would be practically challenging. For simplicity, our mathematical model assumes an idealistic scenario where high-risk individuals in the protected group are unaffected by the pandemic. However, complete protection may not be possible in reality, and prolonged isolation of a large portion of the society would be liable to cause social tensions. Given the existence of high rates of multi-generational families in many countries or settings, implementation of any form of age-targeted strategies, including our strategy, is difficult. In reality, the protective measure would be implemented as comprehensive and detailed lists of guidelines, rather than centralized control of people’s behavior. For example, the protective measure in our framework might be implemented as the advice for elderly people living on their own to have their essentials delivered to their home and to meet their acquaintances outside rather than inside^32^. Since public obedience is always a major issue for such guidelines, assessment based on the proposed approach should be seen as an “upper bound” for the effectiveness of age-diversified control schemes.

Third, our analyses assume lifelong immunity after recovery for simplicity. However, how long immunity against severe acute respiratory syndrome-coronavirus 2 (SARS-CoV-2) lasts or whether we can develop sufficient immunity against SARS-CoV-2 in the first place remains unknown. If sufficient immunity against the virus cannot be developed, herd immunity strategies, in general, would fail. Because of unanswered questions regarding the pathogen, including the ones mentioned above, extreme care should be taken before adopting any form of herd immunity policies. Should a herd immunity policy be adopted despite the expected risks, some protective measures for high-risk individuals are promising methods to achieve both social acceptability and feasibility.

## Supplementary Information

Supplementary material 1

## Data Availability

All data used in this paper are available publicly available. Japanese population composition data is obtained from e-Stat (Statistics of Japan)^16^. The baseline infection fatality ratios are taken from Verity et al.^9^ See supplementary materials for detail.
