# Supplementary material for: Model-based analysis on social acceptability and feasibility of a focused protection strategy against the COVID-19 pandemic
Source: Sci Rep. 2021 Jan 21;11:2003. doi: 10.1038/s41598-021-81630-9 (PMC7820463; doi:10.1038/s41598-021-81630-9)
Supplement: Supplementary file 1 — Supplementary material 1 [file 41598_2021_81630_MOESM1_ESM.pdf]

Supplementary information for “Model-based analysis on social acceptability and feasibility of a focused protection strategy against the COVID-19 pandemic ”

Takashi Akamatsu[1,*], Takeshi Nagae[2,*], Minoru Osawa[3,*], Koki Satsukawa[4], Takara Sakai[5], and Daijiro Mizutani[6]

[1]Tohoku University, Graduate School of Information Sciences, Sendai, Miyagi 980-8579, Japan
[2]Tohoku University, School of Engineering, Sendai, Miyagi 980-8579, Japan
[3]Kyoto University, Institute of Economic Research, Kyoto, Kyoto 606-8501, Japan
[4]Tohoku University, New Industry Creation Hatchery Center, Sendai, Miyagi 980-8579, Japan
[5]Tohoku University, Graduate School of Information Sciences, Sendai, Miyagi 980-8579, Japan
[6]Tohoku University, International Research Institute of Disaster Science, Sendai, Miyagi 980-8572, Japan
[*]TA: akamatsu@plan.civil.tohoku.ac.jp, TN: nagae@tohoku.ac.jp, MO: osawa.minoru.4z@kyoto-u.ac.jp

1 The epidemic model

We consider the basic SIR model[1]. Consider a population that faces a novel infectious disease. The population is categorized into two groups, the protected and the active groups. The former is safely isolated from the virus. The total mass of individuals in the active group is normalized to unity. The relative size of the entire population is $N \geq 1$; thus, $1/N \in (0,1]$ is the share of the active group in the population. Below, S, I, and U denote susceptible, infectious, and recovered individuals, respectively, where $S+I+U=1$. We use U for recovered individuals to avoid confusion with reproduction numbers. The dependence of these quantities on time t is suppressed where no confusion should result.

1.1 Uncontrolled dynamics

The uncontrolled dynamics of the epidemic in the active group is represented by the following equations: $\dot{S} = -\beta SI$, $\dot{I} = \beta SI - \gamma I$, and $\dot{U} = \gamma I$, where $\beta > 0$ is the infection rate and $\gamma > 0$ is the removal rate of infectious individuals, which are constants. The initial condition is $(S(0), I(0), U(0)) = (1-\epsilon, \epsilon, 0)$ where $\epsilon > 0$ is a small number. The average duration of infection is given by $1/\gamma$ per the dynamics; γ equals, say, 20 to 30 days for COVID-19, including the incubation period.

We scale time by the unit of the average duration of infection $1/\gamma$, so that the dynamics reduce into the following dimensionless equations: $\dot{S} = -\mathcal{R}_0 SI$, $\dot{I} = (\mathcal{R}_0 S - 1)I$, and $\dot{U} = I$, where $\mathcal{R}_0 \equiv \beta/\gamma > 0$ is the *basic reproduction number*. We assume $\mathcal{R}_0 > 1$, for which an outbreak occurs from any small $I(0) > 0$ if no control measures are applied. On using this time scale, the area below the epidemic curve $\{I(t) \mid t > 0\}$ coincides with the final number of infected individuals.

The quantity $\mathcal{R}_0 S(t)$ is the *effective reproduction number*, which represents transmissibility at time t for the uncontrolled dynamics. If $\mathcal{R}_0 S(t) > 1$ ($\mathcal{R}_0 S(t) < 1$), then $I(t)$ increases (decreases) in t. This implies that for the uncontrolled case there is a threshold S^* for $S(t)$,

$$S^* = \mathcal{R}_0^{-1}, \tag{1}$$

and $I(t)$ is maximized at t^* such that $S(t^*) = S^*$; the threshold proportion of the cumulative number of infectious individuals to develop herd immunity, or the *herd immunity threshold*, is given by $p^* \equiv 1 - S^* = 1 - \mathcal{R}_0^{-1} \in (0,1)$. The peak number of infectious individuals in the uncontrolled case is given by

$$I_{\text{peak}} \equiv I(t^*) = 1 - \mathcal{R}_0^{-1} - \mathcal{R}_0^{-1} \log \mathcal{R}_0 \tag{2}$$

since $S(t) + I(t) - \mathcal{R}_0^{-1} \log S(t) = S(0) + I(0) - \mathcal{R}_0^{-1} \log S(0) \approx 1$ for all t. As is well known, the final number of infected individuals U_0^* in the uncontrolled scenario is the unique solution for $U_0^* = -\mathcal{R}_0^{-1} \log\left(1 - U_0^*\right)$.

1.2 Controlled dynamics

We assume that we have full control over the social activity level in the active group so that only a time-dependent fraction $\alpha(t) \in [0,1]$, of active individuals can interact with others. The controlled epidemic dynamics become $\dot{S} = -\mathcal{R}_0\alpha SI$, $\dot{I} = (\mathcal{R}_0\alpha S - 1)I$, and $\dot{U} = I$. The effective reproduction number for the controlled dynamics is given by $\mathcal{R}(t) \equiv \mathcal{R}_0\alpha(t)S(t)$. With a control threshold I_c, the proposed adaptive control is as follows (see the bottom panel of Figure 1):

$$\alpha(t) = \begin{cases} 1 & \text{if } I(t) < I_c \quad \text{(no control)}, \\ (\mathcal{R}_0 S(t))^{-1} & \text{if } I(t) = I_c. \end{cases} \tag{3}$$

Under the above control, $\mathcal{R}(t) = \mathcal{R}_0\alpha(t)S(t)$ satisfies

$$\mathcal{R}(t) \begin{cases} > 1 & \text{if } S(t) \geq S^* \text{ and } I(t) < I_c & (0 \leq t < T^*), \\ = 1 & \text{if } S(t) \geq S^* \text{ and } I(t) = I_c & (T^* \leq t \leq T^{**}), \\ < 1 & \text{if } S(t) < S^* & (t > T^{**}), \end{cases} \tag{4}$$

where T^* is the initial time the number of infectious individuals $I(t)$ reaches I_c and T^{**} is the time the active group acquires herd immunity, i.e., $S(T^{**}) = S^*$ (see Figure 1). After T^{**}, the outbreak in the active group proceeds toward the end without any control. Standard final size arguments give the final size of the epidemic in the active group. Under the assumed SIR model, we have the identity

$$S(t) + I(t) - \mathcal{R}_0^{-1}\log S(t) = S(T^{**}) + I(T^{**}) - \mathcal{R}_0^{-1}\log S(T^{**}) \tag{5}$$

at any t after T^{**}. The initial conditions at $t = T^{**}$ are $S(T^{**}) = S^* = \mathcal{R}_0^{-1}$ and $I(T^{**}) = I_c$, provided that $I_c \leq I_{\text{peak}}$, whereas the terminal conditions for $t \longrightarrow \infty$ are $I(\infty) = 0$ and $S(\infty) + U(\infty) = 1$. Plugging these into (5), the *final size* $U^* \equiv U(\infty)$ of the epidemic in the active group under the proposed control policy is given as the solution for the following equation:

$$U^* = 1 - v - \mathcal{R}_0^{-1}\log(1 - U^*) = I_{\text{peak}} - I_c - \mathcal{R}_0^{-1}\log(1 - U^*), \tag{6}$$

where $v \equiv \mathcal{R}_0^{-1} + I_c + \mathcal{R}_0^{-1}\log\mathcal{R}_0$ is the value of the right hand side of (5). By the implicit function theorem regarding (6), we observe

$$\frac{\mathrm{d}U^*}{\mathrm{d}I_c} = \frac{1 - U^*}{U^* - p^*}, \tag{7}$$

implying that U^* is increasing in I_c because $U^* = 1 - S(\infty) > 1 - S^* = 1 - \mathcal{R}_0^{-1} = p^*$.

The protection of high-risk individuals can be lifted at some $T^{***} \gg T^{**}$ where $I(T^{***})$ is sufficiently small so that $U(T^{***}) \approx U^*$. After the protection measure is lifted at time T^{***}, the size of the population becomes N. Then, no second outbreak can occur if $U^* \geq p^*N$, which implies (6) where we denote $A \equiv U^*$.

Plugging the minimum final size $U^* = p^*N$ into (6), we obtain

$$I_{\min} = I_{\text{peak}} - p^*N - \mathcal{R}_0^{-1}\log\left(1 - p^*N\right), \tag{8}$$

provided that $1 - p^*N > 0$, or $n \equiv 1/N > p^*$.

The above derivations for $I_{\min}$ assume that $I_c \leq I_{\text{peak}}$; $I_{\min} > I_{\text{peak}}$ indicates the proposed control is infeasible because we cannot increase U^* by increasing I_c beyond the uncontrolled peak level I_{peak} (see the top panel Figure 1). Figure S1 shows the final size U^* as a function of I_c, which indicates that $A = U^*$ is an increasing function of I_c so long as $I_c \leq I_{\text{peak}}$. We also have $U^* > p^*$ for any $I_c > 0$. However, U^* can not be increased beyond the uncontrolled case U_0^*.

2 Data

2.1 Computation of θ and ϕ

We adopt the age-specific infection hospitalization ratio (IHR) and infection mortality ratio (IFR) provided in, respectively, Table 3 and 1, respectively, of[2]. Other estimates for IFR and IHR can be found in[3], but they yield similar results. We weight-average these ratios by the population composition of Japan[4] to obtain θ and ϕ as (discrete) functions of the threshold age $a^* \in \{5, 10, 15, \ldots, 55, 60, 65, \ldots\}$, up to the availability of the population composition data. Figure S2 shows the computed profiles of θ and ϕ against a^* for Japan. The computed profiles for Great Britain are shown as reference, where the population composition data for Great Britain is obtained from[5]. For both countries, θ and ϕ are increasing in a^*. Because of Japan's aging population, the curves for Japan are steeper than those for Great Britain. We note that the adopted IFR by[2] was obtained by correcting the case fatality ratios (CFR) for under-ascertainment; as a result, the IFR equals roughly half the CFR for Wuhan data, thereby the average ascertainment ratio is approximately 0.5. Our assumption in the main text of a 1/10 times smaller IFR/IHR can thus loosely be interpreted as an assumption that there are 20 times more infections than confirmed cases.

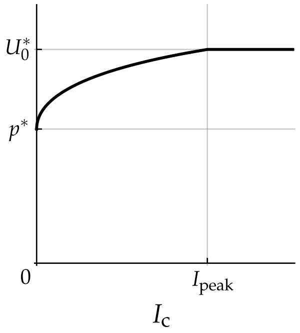

Figure S1. The final number of infected individuals U^* as a function of I_c under a given $\mathcal{R}_0$ where U_0^* denotes the uncontrolled final number. The minimum level for I_c, I_{min}, is the solution for $U^* = p^* N$, which exists only if $p^* N \leq U_0^*$.

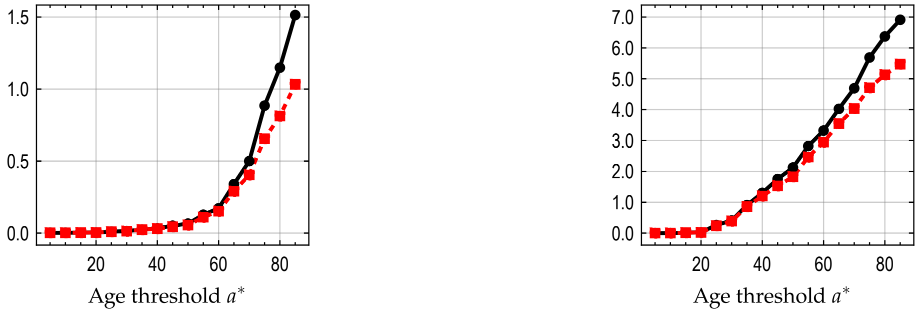

(a) Infection fatality ratio ϕ (%)

(b) Infection hospitalization ratio θ (%)

Figure S2. Infection hospitalization ratio θ and infection fatality ratio ϕ for the active group with different age thresholds a^*. The active group comprises individuals of age $< a^*$. The black solid curve represents Japan, whereas the red dashed curve represents the Great Britain as a reference. As Japan has a larger aging population than Great Britain, both θ and ϕ for Japan rise faster with a^* than those for Great Britain.

2.2 Healthcare service capacity μ

The baseline per capita service capacity for the Japanese healthcare system is set to be $\mu = 3.1 \times 10^4 / 1.26 \times 10^8 = 2.5 \times 10^{-4}$ following a report by the Ministry of Health, Labor and Welfare, which states that the country will prepare 31,077 hospital beds dedicated to the treatment of COVID-19 cases[6].

2.3 Feasibility in terms of the critical care service capacity

Results become similar when we consider an effective capacity on the basis of the ICU service capacity instead of hospitalization. The proportion of individuals who need critical care, which we denote by θ', is reported to be around 10~20% among hospitalized individuals with COVID-19[3,7]. As a conservative assumption, we let $\theta' = 0.2 \times \theta$ where θ is the IHR. The total ICU and high care unit (HCU) bed count for Japan is around 1.7×10^4, or $\mu' \equiv 1.3 \times 10^{-4}$ per capita[8]. These numbers imply $I'_{max} \equiv \mu'/\theta' = 1.3 \times 10^{-4}/(0.2 \times n\theta) = 6.5 \times 10^{-4}/(n\theta)$, which is more than twice greater than $I_{max} = 2.5 \times 10^{-4}/(n\theta)$ in (8). However, the actual effective capacity for critical care would be close to (8), as we acknowledge that (i) the above I'_{max} is an overestimation because the *free* ICU beds per capita must be much fewer than μ' and that (ii) assuming the free ICU beds in the normal setting is probably an underestimation because some special reinforcement measures for COVID-19 must be in place. Because the actual situation regarding the free ICU capacity for COVID-19 cases is unknown, the main text uses the hospitalization capacity in the numerical results provided in the main text.

References

1. Kermack, W. O. & McKendrick, A. G. A contribution to the mathematical theory of epidemics. *Proc. Royal Soc. Ser. A* **115**, 700–721 (1927).

2. Verity, R. *et al.* Estimates of the severity of coronavirus disease 2019: A model-based analysis. *The Lancet Infect. Dis.* DOI: https://doi.org/10.1016/S1473-3099(20)30243-7 (2020).

3. Salje, H. *et al.* Estimating the burden of sars-cov-2 in france. *Science* DOI: https://doi.org/10.1126/science.abc3517 (2020).

4. Ministry of Internal Affairs and Communications. Annual report: Population estimates (2019). https://www.e-stat.go.jp/en/stat-search/files?page=1&layout=datalist&toukei=00200524&tstat=000000090001&cycle=1&year=20200&month=12040604&tclass1=000001011678&result_back=1.

5. for National Statistics, O. Estimates of the population for the UK, England and Wales, Scotland and Northern Ireland (2020). Available from: https://www.ons.gov.uk/peoplepopulationandcommunity/populationandmigration/populationestimates/ (accessed 29 April 2020).

6. Ministry of Health, L. & Welfare. Number of beds for novel coronavirus infections (2020). https://www.mhlw.go.jp/content/10900000/000628694.pdf (accessed 14 May 2020).

7. Remuzzi, A. & Remuzzi, G. COVID-19 and Italy: What next? *The Lancet* (2020).

8. of Intensive Care Medicine, T. J. S. Number of ICU/HCU beds by prefecture (2020). https://www.jsicm.org/news/upload/icu_hcu_beds.pdf (accessed 15 May 2020.
